# An Assessment of the Level of COVID-19 Anxiety among Pregnant Women in Poland: A Cross-Sectional Study

**DOI:** 10.3390/jcm10245869

**Published:** 2021-12-14

**Authors:** Kinga Janik, Urszula Cwalina, Grażyna Iwanowicz-Palus, Mateusz Cybulski

**Affiliations:** 1 Department of Integrated Medical Care, Faculty of Health Sciences, Medical University of Białystok, M. Skłodowskiej-Curie 7A Str., 15-096 Białystok, Poland; mateusz.cybulski@umb.edu.pl; 2 Department of Statistics and Medical Informatics, Faculty of Health Sciences, Medical University of Białystok, Szpitalna 37 Str., 15-295 Białystok, Poland; urszula.cwalina@umb.edu.pl; 3 Department of Development in Midwifery, Faculty of Health Sciences, Medical University of Lublin, Staszica 4/6 Str., 20-081 Lublin, Poland; spupalus@umlub.pl

**Keywords:** anxiety, COVID-19, fear, general anxiety disorder-7 (GAD-7), pregnant, SARS-CoV-2, short health anxiety inventory (SHAI), state-trait anxiety inventory (STAI)

## Abstract

Introduction: The COVID-19 pandemic has caused general anxiety worldwide. Pregnant women are at a much higher risk of developing the infection due to multiple changes that occur in the body during this period. The consequences of the disease can be dramatic not only for the expectant mothers, but also for their unborn children. SARS-CoV-2 infection is generally known to cause serious concerns about future health and life. The data on the severity of COVID-19 pandemic-related anxiety in pregnant women are insufficient. The aim of the study was to assess the level of COVID-19-related anxiety among pregnant women in Poland. Materials and Methods: The study included 173 pregnant women who volunteered for the research. The research was conducted by means of an online diagnostic survey containing an original questionnaire and the following standardized tools: State-Trait Anxiety Inventory (STAI), Short Health Anxiety Inventory (SHAI), and General Anxiety Disorder-7 (GAD-7). Results: Women hospitalised during pregnancy differed statistically significantly in terms of STAI-X1 scores. Primiparas obtained statistically significantly higher SHAI scores than multiparas. Women with higher education had higher SHAI scores. In the GAD-7 scale, 13.3% of respondents obtained a score suggesting a suspected generalised anxiety disorder. Conclusions: Pregnant women are concerned about both developing COVID-19 and the consequences of infection for themselves and their unborn children. The study demonstrated anxiety of varying severity (depending on the tool used). Hospital stay during pregnancy is an additional stressor in expectant women. Further studies are needed to assess the level of COVID-19-related anxiety to assess this phenomenon in Poland in more detail.

## 1. Introduction

The current COVID-19 pandemic is considered to be an example of a natural disaster with such a heavy global health burden, from which more than 236 million people worldwide are suffering and almost 5 million people have died [1]. Poland ranks 16th among the countries with the highest number of coronavirus cases in the world, with 2,918,863 people infected and 75,834 deaths [2].

The COVID-19 pandemic has caused panic and mental health problems around the world [3]. Frequent preventive measures (e.g., washing hands, masking, social distancing, and isolation) during a pandemic obsess people, which increases the risk of psychological damage [4]. Moreover, the pandemic has caused changes in everyday life, which has increased the risk of anxiety and depression [5].

Pregnant women are more vulnerable to any effects of the COVID-19 crisis, which requires action to protect this population [6]. During a pandemic, pregnant women have limited access to primary health care services [7]. Additional factors affecting their mental health are concerns about exposure to COVID-19 and concerns about COVID-19 vaccinations [8]. The prevalence of mental disorders in pregnant women was much higher during the COVID-19 pandemic than in the pre-pandemic period [9,10]. Fear of COVID-19, anxiety, and depression were the most common mental disorders among pregnant women [11,12]. Such disorders were associated with adverse effects of pregnancy, such as preterm labor, low for gestational age, and low birth weight of the newborn [13,14].

While COVID-19 is well-known to cause considerable fear among the general population, there is a little data on the perceived anxiety related to the ongoing COVID-19 pandemic in Poland, particularly among pregnant women. Therefore, the aim of the study was to analyse and assess the symptoms of COVID-19-related anxiety among pregnant women; in particular, to evaluate trait and state anxiety along with health anxiety and generalized anxiety disorder. Additionally, we assessed the impact of selected variables on the severity of anxiety symptoms in the study group. We assumed that the severity of anxiety disorders among pregnant women in Poland would be moderate.

## 2. Materials and Methods

### 2.1. Study Design and Data Collection

The study was conducted from 5 April 2021 to 26 July 2021. The study group included volunteers. Women at various stages of pregnancy were included in the study. We placed a link to the questionnaire, created with the use of a dedicated software (Webankieta) (Get Feedback, Warsaw, Poland) on social media. There were six discussion groups for pregnant women, which gathered 373 women in total. The respondents’ responses were recorded on the platform used and then downloaded as raw data for statistical analysis. The mean time to complete the questionnaire was 23 min.

In addition to female gender and pregnancy, written informed consent was an additional inclusion criterion. Participation in the anonymous study was voluntary. Each participant could withdraw from the study at any time.

There were 589 views on the platform. Considering the number of views, the level of completing the entire questionnaire was 29.33%. A total of 19 questionnaires were not fully completed.

### 2.2. Measures

We used our own questionnaire dedicated for this study. It contained sociodemographic questions and a set of 24 closed questions on the stressors associated with the current epidemiological situation due to SARS-CoV-2 coronavirus infections. Furthermore, we used the following standardised tools: General Anxiety Disorder-7 (GAD-7), Short Health Anxiety Inventory (SHAI), and State-Trait Anxiety Inventory (STAI).

#### 2.2.1. State-Trait Anxiety Inventory (STAI)

The STAI scale is built of two independent subsections. Each part includes 20 items. The first part, STAI X-1, evaluates anxiety as an emotional state at the moment. The second part, STAI X-2, assesses anxiety as a personality trait [15]. The respondent chooses one of the 4 statements for each item. The sum of all the answers for each individuals determines the overall level of anxiety. The score for each subsection is between 20 and 80 points. The higher the total score, the higher the level of anxiety experienced. Suspicion of an anxiety disorders occurs when the sum of the points is 39–40 [16,17].

#### 2.2.2. Short Health Anxiety Inventory (SHAI)

The Short Health Anxiety Inventory (SHAI) is a scale that consists of 18 items and is used to assess anxiety in two aspects: probability of the disease and the negative effects of the disease. Each of the 18 statements contains four options. Respondents choose the one that best describes their emotions over the past 6 months.

The responses are rated on a 4-point Likert scale, where: 0 indicates lack of symptoms, 1—mild symptoms, 2—severe symptoms, and 3—very severe symptoms. The cut-off point for detecting health anxiety is 20 points [18,19].

#### 2.2.3. Generalised Anxiety Disorder Assessment (GAD-7)

The GAD-7 is a seven-point scale that is used to assess the level of anxiety and to assess the risk of developing generalized anxiety disorder (GAD). The questions included in the questionnaire concern the subjective assessment of anxiety, tension, nervousness, the ability to control emotions, the ease of their manifestation, and problems with relaxation The responses are rated on a 3-point Likert scale, where: 0 indicates not at all, 1—several days, 2—more than half the days, and 3—nearly every day. The assessment is based on the past 2 weeks. Scores 5, 10, and 15 are mild, moderate, and severe, respectively. Generalized anxiety disorder is likely if the sum of the points is at least 10 [20].

### 2.3. Procedure and Ethical Considerations

The study was conducted in accordance with the recommendations, and was reviewed and approved by the Ethics Committee of the Medical University of Bialystok (No. APK.002.248.2021). All participants gave a written informed consent in accordance with the Declaration of Helsinki.

### 2.4. Statistical Analysis

Statistica 13.3 (StatSoft Company, Hamburg, Germany) was used for statistical analysis. The analysed variables were of dichotomous, interval, or ordinal nature. The chi-square test was used for dichotomous variables, and basic descriptive statistics for interval results. We used the Mann–Whitney U test to determine statistical significance. The level of statistical significance was set at *p* < 0.05 for each test.

## 3. Results

A total of 173 women participated in the study, which accounted for 49% of women who completed the questionnaire. Detailed sociodemographic characteristics of study participants are presented in Table 1.

The respondents were asked if they were concerned about contracting COVID-19 during pregnancy. It was found that infection with COVID-19 during pregnancy had a significant impact on the answer to this question. Fear of contracting COVID-19 was reported by 30.77% of women with a history of infection and over 50% of women in the group with no such history.

Table 2 summarizes the descriptive statistics of the standardised tools used in the study. A detailed analysis of GAD-7 results showed that the total score indicated anxiety symptoms of varying severity in 71% of respondents. A total of 23 (13.3%) respondents scored at least 10 points, which suggests a suspected generalised anxiety disorder. The mean score obtained by respondents was 13.29 for the SHAI scale. In the GAD-7 scale, most respondents scored between 5 and 9 points. The mean scores obtained in STAI-XI and STAI-X2 were similar, i.e., 42.26 and 40.24, respectively. Details are shown in Table 2.

Primiparas were found to have statistically significantly (*p* = 0.031) higher SHAI scores (M = 14.45, Me = 14) compared to multiparas (M = 12.22, Me = 12). Primiparas were significantly younger. Both study groups were also compared for the following scales: GAD-7 and both STAI subscales. No statistically significant differences were found for these tools (Table 3).

The analysis has shown that women with higher education scored statistically significantly higher in SHAI (*p* = 0.019, M = 14.17, Me = 13) and GAD-7 (*p* = 0.006, M = 6.31, Me = 6). No statistically significant results were found for the other scales (Table 4).

Hospitalised pregnant women scored significantly higher in STAI-X1. No statistically significant differences were found between pregnant women hospitalised during pregnancy and those not requiring hospitalisation in the remaining scales (Table 5).

## 4. Discussion

So far, it has been established that the outbreak of the pandemic increased the level of mental health disorders in the general population [21,22], and it is more visible in women than in men. Some studies report that during catastrophes or major events, pregnant women are more likely to develop mental health problems than in the general population [23,24]. A recent systematic review and meta-analysis [25] assessing the impact of the pandemic on the mental health of pregnant women has shown that the level of mental health disorders in pregnant women is 37%. Our study showed that women who had COVID-19 had lower levels of fear of infection with the SARS-CoV-2 than pregnant women who had not contracted the disease so far. Different results were obtained in another Polish study by Nowak et al. [26]. The authors proved that pregnant women who had been infected with SARS-CoV-2 experienced a higher level of anxiety than those who had not been infected so far.

Our study showed moderate anxiety among pregnant women in the STAI scale. Another study among pregnant women also assessed the level of antenatal anxiety using the STAI scale. Similar findings were obtained in both studies [27]. Similar results in the same scale were also shown in a study in Italy [28]. These findings confirm the conclusions obtained by Sinjari et al. [29], that questionnaires could be useful tools to assess patients’ conditions before the visit to a doctor.

Considering the parity of respondents, we found that primiparas show higher COVID-19-related anxiety than multiparous women, as confirmed by SHAI scores. STAI results reported by Italian researchers also confirmed our hypothesis [28]. Such findings may be due to both the new life situation and the lack of knowledge about pregnancy and coronavirus infection during this special time.

The data on the impact of gestational age on the level of COVID-19-related anxiety among pregnant women are contradictory. In our analysis, we compared the scores obtained in the standardized scales depending on the trimester of pregnancy, but no statistically significant differences were found. Other authors reported higher STAI scores in the first and third trimesters than those obtained in our study [30]. Schubert et al. showed that, on the other hand, STAI scores remained stable throughout pregnancy [31].

We measured the prevalence of anxiety symptoms using the GAD-7 scale. It was found to be high, i.e., 62.5% among pregnant women, including 49% with mild, 10% with moderately severe, and 3.5% with severe anxiety due to COVID-19. Other studies have also shown that pregnant women are much more prone to stress during the COVID-19 pandemic [32].

Our analysis showed that pregnant women with higher education were significantly older and scored statistically significantly higher in both SHAI and GAD-7. Similar findings on the impact of higher education on the increased levels of anxiety have been reported by other authors [32]. It can be assumed that greater awareness of one’s own health negatively affects its loss as a result of a serious illness. The differences in the obtained results can be due to the dynamic nature of the disease and the fact that it is perceived differently around the world.

In this study, the median STAI score for anxiety was 41. A total of 57.23% of the pregnant women scored ≥40. In a similar study involving pregnant women, the median score in the same scale was 37, including a score of ≥40 in 38.2% of participants [33]. Another study among pregnant women showed that the overall prevalence of anxiety symptoms measured with STAI (STAI > 40) was 62.6% [34]. An Italian study found similar levels of COVID-19-related anxiety in pregnant women (68%) [30]. Turkish studies also reported STAI scores above the cut-off for clinically significant symptoms of anxiety [35]. Similar findings in all the above-mentioned studies may be due to the different state of the women (pregnancy) and their concern about the health of their unborn children.

The presented results confirm COVID-19-related anxiety among pregnant women. Its level varies and is related to sociodemographic factors. Due to the negative effects of anxiety and stress on pregnant women and their unborn children, further research is needed on anxiety caused by the COVID-19 pandemic to prevent negative effects and improve the health of the population. Mental health screening for pregnant women should be included in the mandatory prenatal screening to reduce any potential anxiety symptoms.

### Limitations of the Study

The study has certain limitations. The presented results come from an analysis based on a subjective assessment of anxiety symptoms in pregnant women. Although we used scales that are considered sensitive research tools, they are based on subjective feelings and do not include objective criteria of clinical symptoms, which may lead to false-positive results. The number of pregnant women participating in the study is another limitation. The small sample size does not allow for extrapolation of results to the general population of pregnant women in Poland. However, despite these limitations, our findings can be a reference point for further studies assessing the level of COVID-19-related anxiety among pregnant women both in Poland and in the world.

## 5. Conclusions

In conclusions, primiparas showed statistically significantly higher anxiety levels than multiparas. Higher education also contributed to higher scores. Hospital stays during pregnancy contributed to statistically higher STAI-X1 scores. There was no statistically significant relationship between pregnancy trimester and the prevalence of COVID-19. There is a need to continue research on COVID-19-related anxiety among pregnant women in Poland to assess this phenomenon and the causative factors in more detail.

The COVID-19 pandemic has serious consequences. Therefore, in the periods of health crisis, it is imperative to develop research focused on studying the phenomenon and its impact with other variables in order to understand its impact on pregnant women. This can help prevent and address negative mental health effects that can affect the functional status and quality of life of pregnant women.

## Figures and Tables

**Table 1:** Sociodemographic characteristics of respondents.

Sociodemographic Feature		*n*	%
Age (years)	<25	15	8.67%
	25–34	132	76.30%
	≥35	26	15.03%
Education	middle school	1	0.58%
	basic vocational	13	7.51%
	secondary	46	26.59%
	higher	113	65.32%
Marital status	married	150	86.70%
	divorced	3	1.73%
	single	15	8.67%
	unmarried relationship	5	2.89%
Place of residence	urban	139	80.35%
	rural	34	19.65%
Socioeconomic status	very good	39	22.54%
	good	111	64.16%
	moderate	23	13.30%
	poor	0	0.00%
Number of children	0	83	47.98%
	1	65	37.57%
	2	17	9.83%
	3	6	3.47%
	4	1	0.58%
	5	1	0.58%
Parity	primipara	83	47.98%
	multipara	90	52.02%
Trimester	I (1–13 weeks)	16	9.25%
	II (14–26 weeks)	25	14.45%
	III (27–40 weeks)	132	76.30%
History of mental disorders	yes	9	5.20%
	no	164	94.80%
History of psychotropic therapy	yes	10	5.78%
	no	163	94.22%
History of the prevalence of COVID-19	yes	26	15.03%
	no	147	84.97%

**Table 2:** Summary of descriptive statistics of the standardised research tools.

	M	SD	Q_1_	Me	Q_3_	Min.	Max.
SHAI	13.29	6.17	9	13	17	2	40
GAD-7	5.79	3.90	3	5	7	0	21
STAI-X1	42.26	11.20	34	41	50	22	74
STAI-X2	40.25	8.50	34	40	45	23	69

**Abbreviations:** GAD-7—General Anxiety Disorder-7, M—mean, Max.—maximum, Me—median, Min.—minimum, SD—standard deviation, SHAI—Short Health Anxiety Inventory, STAI—State-Trait Anxiety Inventory, Q_1_—lower quartile, and Q_3_—upper quartile.

**Table 3:** Comparison of primiparas and multiparas for SHAI, GAD-7, and STAI.

Tools	Primiparas (*n* = 83)					Multiparas (*n* = 90)					*p*
	M	SD	Q_1_	Me	Q_3_	M	SD	Q_1_	Me	Q_3_	
**SHAI**	14.45	6.81	10	14	18	12.22	5.35	9	12	16	0.031
**GAD-7**	6.45	4.27	4	6	8	5.19	3.43	3	5	7	0.066
**STAI-X1**	43.49	12.01	35	41	53	41.12	10.33	33	41	47	0.218
**STAI-X2**	40.35	8.24	34	41	46	40.16	8.78	34	40	44	0.753

**Abbreviations:** GAD-7—General Anxiety Disorder-7, M—mean, Me—median, *p*—*p*-value, SD—standard deviation, SHAI—Short Health Anxiety Inventory, STAI—State-Trait Anxiety Inventory, Q_1_—lower quartile, and Q_3_—upper quartile.

**Table 4:** Comparison of women with and without higher education for the following tools: SHAI, GAD-7, and STAI.

Tools	Higher Education (*n* = 113)					No Higher Education (*n* = 60)					*p*
	M	SD	Q_1_	Me	Q_3_	M	SD	Q_1_	Me	Q_3_	
**SHAI**	14.17	6.15	10	13	18	11.63	5.93	7.5	12	15.5	0.019 *
**GAD-7**	6.31	3.96	4	6	8	4.82	3.62	2	4	7	0.006 *
**STAI-X1**	41.87	11.19	34	40	48	43	11.27	37	41	52	0.369
**STAI-X2**	40.21	8.75	33	40	44	40.32	8.08	35.5	40	45.5	0.777

**Abbreviations:** GAD-7—General Anxiety Disorder-7, M—mean, Me—median, *p*—*p*-value, SD—standard deviation, SHAI—Short Health Anxiety Inventory, STAI—State-Trait Anxiety Inventory, Q_1_—lower quartile, Q_3_—upper quartile, and *—statistically significant.

**Table 5:** The impact of hospital stays during pregnancy on the rating of anxiety.

Tools	Hospitalised during Pregnancy (*n* = 93)					Not Hospitalised during Pregnancy (*n* = 80)					*p*
	M	SD	Q_1_	Me	Q_3_	M	SD	Q_1_	Me	Q_3_	
**SHAI**	12.77	5.52	10	12	15	13.88	6.84	8.5	13.5	18	0.215
**GAD-7**	5.76	3.91	3	6	7	5.82	3.90	3	5	7	0.951
**STAI-X1**	44.14	11.55	37	41	52	40.07	10.42	32	39	46.5	0.019 *
**STAI-X2**	39.55	8.02	34	40	44	41.06	9.01	34.5	40	45	0.459

**Abbreviations:** GAD-7—General Anxiety Disorder-7, M—mean, Me—median, *p*—*p*-value, SD—standard deviation, SHAI—Short Health Anxiety Inventory, STAI—State-Trait Anxiety Inventory, Q_1_—lower quartile, Q_3_—upper quartile, and *—statistically significant.

## Data Availability

Data are available upon reasonable request.
